# Fucoidan-Mediated Anisotropic Calcium Carbonate Nanorods of pH-Responsive Drug Release for Antitumor Therapy

**DOI:** 10.3389/fbioe.2022.845821

**Published:** 2022-04-13

**Authors:** Pei Wang, Fei Tong, Jun Luo, Zhihua Li, Junchao Wei, Yuangang Liu

**Affiliations:** ^1^ School of Stomatology, Nanchang University, Nanchang, China; ^2^ Jiangxi Province Key Laboratory of Oral Biomedicine, Nanchang, China; ^3^ Jiangxi Province Clinical Research Center for Oral Diseases, Nanchang, China; ^4^ Institute of Pharmaceutical Engineering, Huaqiao University, Xiamen, China

**Keywords:** anisotropic nanorods, biocompatibility, control release, chemotherapy, polysaccharide

## Abstract

The shape of nanoparticles can determine their physical properties and then greatly impact the physiological reactions on cells or tissues during treatment. Traditionally spherical nanoparticles are more widely applied in biomedicine but are not necessarily the best. The superiority of anisotropic nanoparticles has been realized in recent years. The synthesis of the distinct-shaped metal/metal oxide nanoparticles is easily controlled. However, their biotoxicity is still up for debate. Hence, we designed CaCO_3_ nanorods for drug delivery prepared at mild condition by polysaccharide-regulated biomineralization in the presence of fucoidan with sulfate groups. The CaCO_3_ nanorods with a pH sensitivity–loaded antitumor drug mitoxantrone hydrochloride (MTO) showed excellent antitumor efficacy for the HeLa cells and MCF-7 cells *in vitro*. We believe that anisotropic nanoparticles will bring forth an emblematic shift in nanotechnology for application in biomedicine.

## 1 Introduction

Control over the structure, shape, size, and morphology of nanomaterials is an important fundamental goal of bioscience owing to their important roles in determining the properties. In particular, spherical nanoparticles such as nanospheres and liposomes are most broad and widely applied as drug carriers due to easy accessibility (Yang et al., 2018). Nevertheless, spherical nanoparticles are not necessarily in optimal shape for nanocarriers. For instance, Banerjee et al. found that the order of cellular uptake efficiency for the nanoparticles was rod > disc > sphere (Banerjee et al., 2016). Agarwal et al. found maximal accumulation of nanoparticles with disc shape rather than nanospheres in an *in vitro* tumor tissue model (Agarwal et al., 2015). Actually, red blood cells and many representative pathogens in human bodies have distinct shapes. Nowadays, the superiority of nonspherical nanoparticles attracts more and more attention from researchers due to their distinctive physicochemical properties and promising applications in biomedicine (Xie et al., 2017; Ding et al., 2018). The distinct-shaped nanoparticles exhibit different properties affecting their behavior *in vivo* during the delivery and reaction with cells. In addition to impacting the internalization of nanoparticles, blood circulation time and biodistribution also are discrepant for nanomedicine because macrophage uptake during the blood circulation is usually clear circulating nanoparticles (Copp et al., 2014; Liu et al., 2018). In contrast to spherical nanoparticles dependent on clathrin- and caveolin-mediated endocytosis, uptake of rod-shaped nanoparticles mainly based on clathrin-mediated endocytosis by macrophages has been found to be lower (Li et al., 2016). Special-shaped nanoparticles may improve the target for tumor tissues (Agarwal et al., 2015), but there is no report showing only targeting tumor cells or normal cells due to the shape of the nanoparticle.

Over the past years, the nanoparticle shapes of rod (Nakayama et al., 2015), belt (Talloj et al., 2020), wire (Hu et al., 2021), sheet (Mutalik et al., 2022), bipyramid (Yougbaré et al., 2021), triangle (Kuwahara et al., 2020), hexagon (Yong et al., 2021), disc (Chen et al., 2020), cube (Wang et al., 2021), octahedron (Park et al., 2018), tripod (Feng et al., 2020), star (Spedalieri et al., 2021), thorn (Li et al., 2020a), and tetrapod (Qiu and Yang, 2007) have been synthesized for different applications. For example, gold nanorods have been widely used in photothermal therapy because of their surface plasmon peaks in the near-infrared laser in comparison to nanospheres (Darwish et al., 2020). In particular, the shape-effect research studies are mainly dependent on metal or metal oxide nanoparticles such as gold and silver because they are easily controlled (Zhu et al., 2016; Chakraborty and Parak, 2019). However, the cytotoxicity of metallic nanoparticles has to be considered. In addition, the biodegradable organic nanoparticles with anisotropy are usually difficult to synthesize (Yang et al., 2018). Hence, the biocompatible nanoparticles such as mesoporous silica nanoparticles (Kankala et al., 2020), hydroxyapatite nanoparticles (Li et al., 2019; Tan et al., 2020), and calcium carbonate (CaCO_3_) nanoparticles (Chaudhary and Maiti, 2019) were considered for application in biomedicine.

Calcium carbonate (CaCO_3_), one of the most abundant minerals, has attracted widespread attention in the biomedicine field owing to its low cost, biocompatibility, biodegradability, and pH-sensitivity (Maleki Dizaj et al., 2015; Dong et al., 2016; Zheng et al., 2021). Hence, the CaCO_3_ nanoparticles were used as drug/gene delivery vehicles. Lu et al. designed CaCO_3_/pneumolysin antigen delivery systems by physical absorption to induce cellular immunity for immunotherapy (Lu et al., 2021a). Chen et al. successfully synthesized polyethyleneimine-modified CaCO_3_ nanoparticles delivering p53 gene for gene therapy (Lu et al., 2021a). In particular, the CaCO_3_ nanoparticles keep steady under a neutral environment and would be decomposed into Ca^2+^ and CO_2_ in acidic pH. In another work, we constructed poly-L-ornithine/fucoidan-coated CaCO_3_ particles with pH-controlled doxorubicin release for cancer therapy (Wang et al., 2018a). At present, the synthesis process of CaCO_3_ nanoparticles is carried out mainly through ammonium bicarbonate (NH_4_HCO_3_) continuously diffusing into the calcium chloride (CaCl_2_) solution. Moreover, the biomineralization method due to environmental friendliness and wide availability received tremendous attention in recent years (Sugawara-Narutaki, 2013; Lu et al., 2021b; Yang et al., 2021). Therein, the cooperative involvement of macromolecules during the crystallization of CaCO_3_ nanoparticles is a benign strategy, such as polyacrylic acid (Nakayama et al., 2015), polyacrylamide (Yu et al., 2006), chitin (Ehrlich, 2010), and silk fibroin (Cao, 2008). Therein, fucoidan, as a natural polysaccharide, is a dramatic candidate for biomedical applications due to its unique properties such as biocompatibility and less immunogenicity (Lu et al., 2017; Hsu et al., 2018).

**FIGURE 1:**
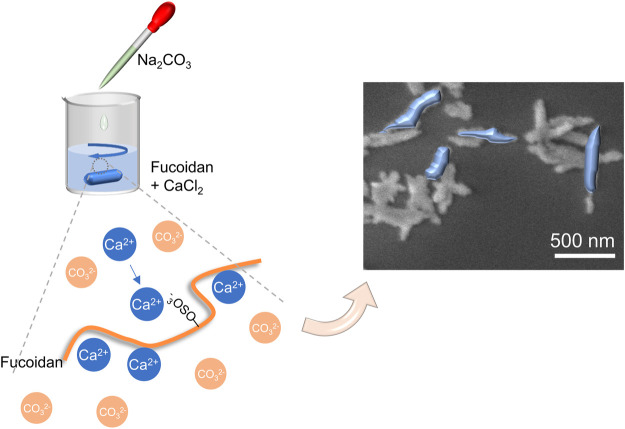
Schematic diagram of the synthesis of CaCO_3_ nanorods.

In this work, inspired by biomineralization, we develop anisotropic calcium carbonate nanoparticles having a rod structure through the coprecipitation method mediated by fucoidan (Figure 1). Importantly, the mechanism of CaCO_3_ nanorods (NRs) with different aspect ratios was tested by changing the concentration of fucoidan. Moreover, we applied CaCO_3_ nanorods in the field of biomedicine after loading with an antitumor drug MTO owing to its degradability in the acidic tumor environment, which can release the drugs quickly and achieve the pH-response drug release. As a result, efficient tumor regression was achieved, suggesting a new avenue for the exploitation of safe and effective therapeutic nanorods.

## 2 Materials and Methods

### 2.1 Materials

All the reagents and chemicals were acquired commercially. Sodium carbonate and calcium chloride were purchased from Sinopharm Chemical Reagent Co., Ltd. (Shanghai, China). Fucoidan (Mw = 200–400 kDa) was purchased from Jiejing Group (Shandong, China). Mitoxantrone hydrochloride (MTO) was obtained from Meilun Biotech Co., Ltd., (Dalian, China). Penicillin, streptomycin, fetal bovine serum (FBS), and Dulbecco’s modified Eagle medium (DMEM) were obtained from Biological Industries Ltd., (Hertzliya Pituach, Israel). The HeLa cells, MCF-7 cells, L929 cells, and C2C12 cells were acquired from the Type Culture Collection of Chinese Academy of Sciences (CAS) (Shanghai, China). MTT Cell Proliferation, acridine orange (AO)/ethidium bromide (EB) Cell Live/dead Kit, and DAPI Staining Solution were obtained from KeyGen Biotech Co., Ltd. (Nanjing, China).

### 2.2 Synthesis of Calcium Carbonate NRs

The CaCO_3_ NRs were synthesized by utilizing a biomimetic method at room temperature. Briefly, calcium chloride (0.21 g) and sodium carbonate (0.22 g) were dissolved in 100 ml of water (0.02 M). Then, fucoidan was also dissolved in water at diverse concentrations (5, 10, 20, 30, 40, and 50 mg/ml). Furthermore, fucoidan (10 ml) was added to the calcium chloride solution (10 ml) and stirred for 1 h. Subsequently, sodium carbonate solution (10 ml) was added dropwise to the mixed solution of fucoidan and sodium carbonate to synthesize CaCO_3_ NRs. Eventually, the products were collected, washed, and suspended in water.

### 2.3 Characterization of Calcium Carbonate NRs

We used the dynamic light scattering (DLS) instrument (ZetaPALS; Malvern Instruments Co., Ltd.) to measure the nanoparticle size distribution and zeta potential of the designed CaCO_3_ NRs. The surface morphology was observed by using the scanning electron microscope (SEM, Hitachi S-4800) and transmission electron microscope (TEM, Hitachi H-7650). The powder X-ray diffraction (PXRD, Bruker AXS D8 Advance) analysis of the copper-impregnated and naked CaCO_3_ NRs was carried out from 10° to 80° using Cu–Kα radiation. The characteristic groups on the CaCO_3_ nanorods were analyzed by using Fourier transform infrared spectroscopy (FT-IR, Thermo Scientific Nicolet iS 50). The sample was prepared using a KBr pellet method.

### 2.4 Drug Loading and Encapsulation Efficiency Measurement

Mitoxantrone hydrochloride (MTO) was loaded with the CaCO_3_ NRs using the following procedure. First, 5 mg of CaCO_3_ NRs were dispersed in 25 ml phosphate-buffered solution (PBS) and then 5 mg of MTO was added for loading. After stirring overnight, the product defined as the MC NRs was centrifuged and washed twice with water. Furthermore, the supernatant concentration of MTO was detected by UV–Vis spectroscopy (TU-1810, PERSEE) at 663 nm and calculated with the calibration curve of MTO. Finally, the loading amount and encapsulation efficiency of MTO were calculated according to the following formulas:

$$\text{L}\text{o}\text{a}\text{d}\text{i}\text{n}\text{g}\;\text{a}\text{m}\text{o}\text{u}\text{n}\text{t}\left(\%\right)=\left(MTO_{t}-\;MTO_{s}\right)/MC\;NRs\times 100$$

$$\text{E}\text{n}\text{c}\text{a}\text{p}\text{s}\text{u}\text{l}\text{a}\text{t}\text{i}\text{o}\text{n}\;\text{e}\text{ff}\text{i}\text{c}\text{i}\text{e}\text{n}\text{c}\text{y}\left(\%\right)=\left(MTO_{t}-\;MTO_{s}\right)/\left(MTO_{t}\right)\times 100$$

where MTO_t_ is the total weight of MTO, MTO_s_ is the supernatant weight free MTO, and MC NRs is the weight of MC NRs.

### 2.5 Mitoxantrone Hydrochloride Release From MC NRs *in vitro*

MC NRs (5 mg) were moved into the dialysis bag, and then 20 ml of PBS was added for MTO release. Then, PBS was moved in a shaker kept at 37°C and 100 rpm for 72 h. PBS (2 ml) was removed for the measure at various periods using UV–Vis spectroscopy according to the calibration curve of MTO, and then 2 ml of fresh PBS was supplemented. All the measurements were carried out in triplicate.

### 2.6 Biocompatibility Assay of Calcium Carbonate NRs

#### 2.6.1 Cytotoxicity Assay

The cytotoxicity assay of the CaCO_3_ NRs was determined by methyl thiazolyl tetrazolium (MTT) for the L929 cells. First, L929 cells and C2C12 cells with 6 × 10^3^ cells were seeded into a 96-well plate. After 24 h, DMEM was removed and 100 μl of fresh medium containing CaCO_3_ NRs at different concentrations was added for coincubating for 24 h. Subsequently, the MTT reagent (10 μl) was added to each well for coincubating for 4 h. Finally, DMEM was removed and 150 μl of DMSO was added into a 96-well plate for measuring the absorbance by using a microplate reader (Varioskan Flash 1,510, Thermo Fisher Scientific). The cell viability rate was calculated by the following formula:

$$\text{C}\text{e}\text{l}\text{l}\;\text{v}\text{i}\text{a}\text{b}\text{i}\text{l}\text{i}\text{t}\text{y}\;\text{r}\text{a}\text{t}\text{e}\left(\%\right)=\left(OD_{\mathrm{treated}}\;-\;OD_{\mathrm{free}}\right)/\left(OD_{\mathrm{control}}\;-\;OD_{\mathrm{free}}\right)\times 100$$

where OD_treated_ is the absorbance of CaCO_3_ NRs, OD_free_ is the absorbance of only DMSO, and OD_control_ is the control group.

#### 2.6.2 Hemolysis Test

To evaluate the hemolysis of CaCO_3_ NRs, CaCO_3_ NRs were incubated with normal saline and then added 0.2 ml diluted rabbit blood for 60 min at 37°C. Furthermore, we measured the supernatant absorbance at 545 nm after centrifugation. The absorption of positive and negative control experiments was incubated with H_2_O and normal saline, respectively. The hemolysis rate was calculated by the following formula:

$$\text{H}\text{e}\text{m}\text{o}\text{l}\text{y}\text{s}\text{i}\text{s}\;\text{r}\text{a}\text{t}\text{e}\left(\%\right)=\left(OD_{\mathrm{treated}}\;-\;OD_{\mathrm{negative}}\right)/\left(OD_{\mathrm{positive}}\;-\;OD_{\mathrm{free}}\right)\times 100$$

where OD_treated_ is the absorbance of CaCO_3_ NRs, OD_negative_ is the absorbance of normal saline, and OD_positive_ is the absorbance of H_2_O.

### 2.7 Cellular Uptake Study

The MCF-7 cells and HeLa cells were seeded at the cover glass in the 24-well plate for 24 h. Then, after coincubation with MC NRs for 2 and 4 h, the cover glass was washed three times with PBS. After staining with 4′,6-diamidino-2-phenylindole (DAPI), the cellular uptake images were observed by using a confocal laser scanning microscope (CLSM, Leica TCS SP5).

### 2.8 Antitumor Assay *in vitro*

For the antitumor assay *in vitro*, HeLa cells and MCF-7 cells were seeded into 96-well plates. Then, DMEM was removed and 100 μl of fresh DMEM containing MTO and MC NRs at diverse concentrations of 1, 2, 3, 5, and 10 μg/ml was added. Other details were the same as described in Section 2.6.1.

Apoptosis of the cancer cells was observed using the AO/EB Kit. The HeLa cells and MCF-7 cells were seeded into a 6-well plate and incubated for 24 h. The media were replaced with 1, 2, 3, 5, and 10 μg/ml of samples (MTO and MC NRs). After 24 h of incubation, the HeLa cells and MCF-7 cells were washed with PBS. Then, AO and EB were added and observed using a fluorescence microscope (Zeiss AXIO Observer Z1).

### 2.9 Statistical Analysis

All the data were expressed as the mean ± standard deviation (SD, n ≥ 3). The statistical significance was calculated *via* one-way analysis of variance (ANOVA) followed by Tukey’s test. A *p*-value of <0.05 was considered statistically significant (*p < 0.05, **p < 0.01, ***p < 0.001).

## 3 Results and Discussion

### 3.1 Physicochemical Characterizations

**FIGURE 2:**
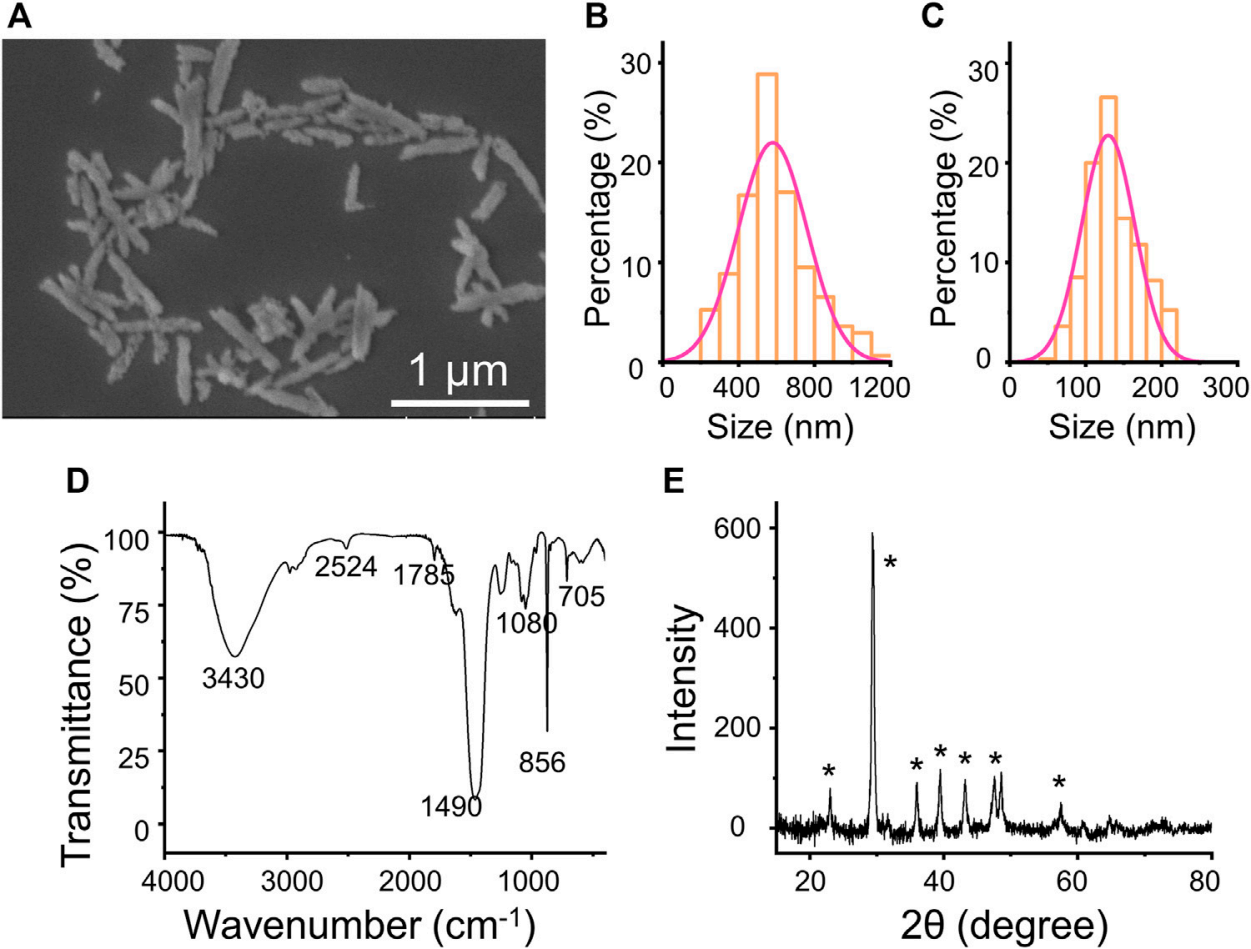
**(A)** SEM images of the CaCO_3_ NRs. **(B)** length and **(C)** width statistical chart of the CaCO_3_ NRs. **(D)** FT-IR spectrum **(E)** XRD of CaCO_3_ NRs.

The rod anisotropic CaCO_3_ nanoparticles have been successfully prepared by utilizing a kind of natural polysaccharide, fucoidan, as a crystal mediator determined by the crystallographic growth direction and stabilizing agent prevented agglomeration of particles. The procedure for the preparation of CaCO_3_ NRs is shown in Figure 1. Initially, Ca^2+^ was concentrated to fucoidan due to electrostatic attraction and the reaction between the sulfuric ester group and Ca^2+^ in the mixed solution at room temperature. Then, Na_2_CO_3_ aqueous solution was added dropwise for the crystallization of CaCO_3_ NRs. During the process, the turbidity of the solution increased, which suggested the synthesis of crystalline CaCO_3_ nanoparticles. The resultant CaCO_3_ particles revealed a rod-shaped morphology at a submicrometer scale as observed by the SEM (Figure 2A). It should be noted that the monodisperse CaCO_3_ nanorods were mediated with a relatively narrow distribution in size as shown in Figure 2B and Figure 2C. The histograms of length and width of the CaCO_3_ NRs were measured by counting more than 300 samples pictured in the SEM. The average length and width of the CaCO_3_ NRs were 579 ± 151 nm and 130 ± 35 nm, respectively, suggesting that the aspect ratio was 4.5. In addition, the quality content of S, Ca, and C from the CaCO_3_ NRs was 0.4, 55.3, and 44.3% (Supplementary Figure S2), respectively. Moreover, the effects of fucoidan concentration on the size and morphology of CaCO_3_ NRs were examined. The morphologies of the CaCO_3_ nanorods are analogous with the distinct concentration of fucoidan (Supplementary Figure S1). The size of the nanorods was decreased with increasing the concentration of fucoidan from 5 to 30 μg/ml and then increasing from 40 to 50 μg/ml (Table 1). On the basis of these results, it is suggested that fucoidan not only stabilizes the anisotropic CaCO_3_ nanorods but the size of nanorods is also sensitive to the fucoidan concentration.

**TABLE 1:** Average length and width of the calcium carbonate nanorods prepared with different fucoidan concentrations.

Fucoidan (µg/ml)	5	10	20	30	40	50
Length (nm)	1,104 ± 340	792 ± 259	790 ± 226	570 ± 151	725 ± 223	855 ± 349
Width (nm)	462 ± 102	252 ± 45	204 ± 31	130 ± 35	170 ± 42	314 ± 71
Aspect ratio	2.4	3.1	3.9	4.5	4.3	2.7

Furthermore, the crystalline phase of the nanocrystals was identified as aragonite by FT-IR spectroscopy (Figure 2D) and XRD measurement (Figure 2E). The transmittance peaks in the FT-IR spectrum at 705, 856, and 1,080 cm^−1^ are attributed to the CaCO_3_ aragonite form (Yu et al., 2006). In addition, the peaks at 856 cm^−1^ and 705 cm^−1^ are ascribed to the vibration of the out-of-plane bending and in-plane bending of CO_3_^2−^ in aragonite, respectively. In addition, all the peaks in the XRD pattern (Figure 2E) are characteristic of aragonite. The diffraction peaks of the CaCO_3_ nanorods from the XRD (labeled with the symbol ∗) could be indicated to the aragonite CaCO_3_ accordingly (JCPDS Card no. 05-0453).

### 3.2 *In vitro* Mitoxantrone Hydrochloride Release From MC NRs

**FIGURE 3:**
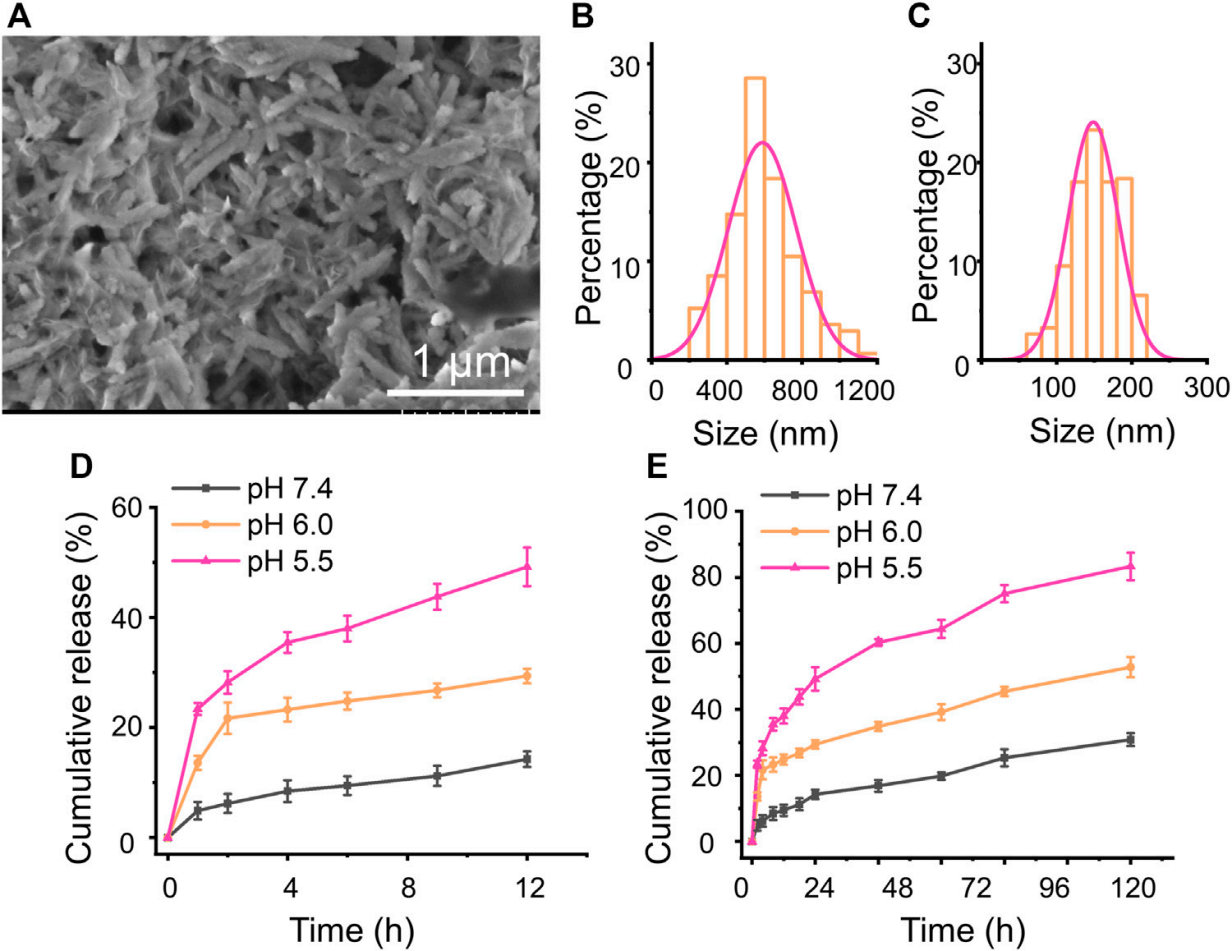
**(A)** SEM images of the MC NRs. **(B)** length and **(C)** width statistical chart of the MC NRs. *In vitro* MTO cumulative release from the MC NRs in pH 7.4 and 6.0 and pH 5.5 PBS for **(D)** 12 h and **(E)** 72 h.

MTO, as a broad anticancer drug, can intercalate into the DNA or RNA through hydrogen bonding to induce cross-links and strand breaks and interfere with the stabilization of DNA topoisomerase II cleavable complex (Duan et al., 2013; Wang et al., 2019). Herein, the MTO was used to appraise the loading amount of the previously prepared CaCO_3_ NRs and observed the pH-sensitive release behavior. To prepare the MTO-loaded CaCO_3_ NRs (MC NRs), MTO was quickly added into the prepared PBS containing CaCO_3_ NRs and after stirring overnight, the unloaded MTO was removed by centrifugation. In terms of the calibration curves of MTO (Supplementary Figure S3), the loading amount and entrapment efficiencies of the MC NRs were calculated as 34.5 ± 2.8% and 52.7 ± 1.9%, respectively. After loading the drug, the characteristics of the MC NRs were observed from the SEM (Figure 3A), which intuitively presented a smoother surface. Moreover, the histograms of length (Figure 3B) and width (Figure 3C) of the MC NRs were measured by counting 300 samples pictured in the SEM, which showed that the MTO-loaded nanoparticles are larger than those of the unloaded ones, suggesting that MTO was loaded into the CaCO_3_ NRs. The average length and width of the MC NRs were 590 ± 182 nm and 149 ± 33 nm, respectively, suggesting that the aspect ratio of the nanorods was 4.0.

As we all know, CaCO_3_ can be disintegrated and release Ca^2+^ and CO_2_ at low pH. Afterward, a release assay using PBS with pH 7.4, 6.0, and 5.5 simulating the intracorporeal conditions was carried out, drawing the MTO cumulative release profile from the MC NRs. The classic dialysis method with time intervals was used for 72 h. Initially, burst MTO release from the MC NRs within 12 h was observed at neutral and acidic pH (Figure 3D), which might be attributed to the water-induced dissolution. In addition, the cumulative release rate dramatically increased with a decrease of pH from 7.4 to 5.5, as PBS with pH 5.5 presented the most drastic MTO release (Figure 3D). In particular, merely 17% of the drug was released over 12 h in PBS of pH 7.4, suggesting that the MTO stably loading with the CaCO_3_ NRs was well-realized. In addition, diminishing the pH value to 5.5 increased the drug release in the MC NRs to approximately 50% at 12 h, proving the pH-responsive property of the CaCO_3_ NRs (Ferreira et al., 2020). It is an advantage for the MC NRs to serve as a multifunctional drug delivery system in biomedical applications. Notably, the CaCO_3_ NRs with the nature of acid-triggered decomposition could be appropriate for long blood circulation under the physiological pH and quick release within the acid tumor microenvironment and lysosomes.

### 3.3 Biocompatibility Study

**FIGURE 4:**
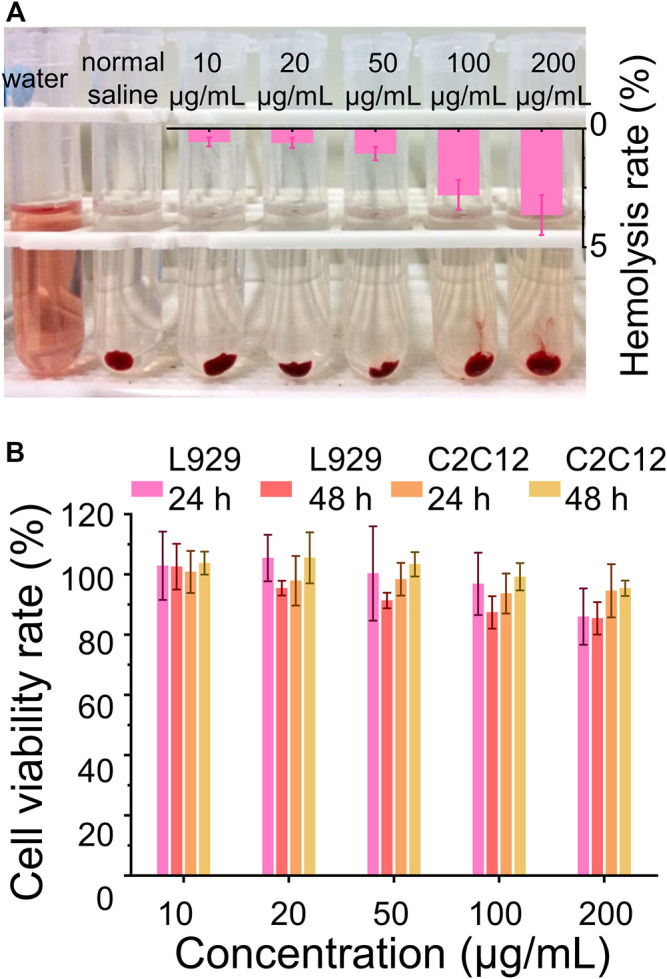
Biocompatibility assay. **(A)** Hemolysis assay after the treatment with different concentrations of the MC NRs. **(B)** Cell viability rate of the L929 cells and C2C12 cells treated with CaCO_3_ NRs for 24 and 48 h.

Biocompatibility is one of the important requirements for biomaterials, which should neither adversely affect normal cells nor destruct their normal balance in clinical applications (Janeesh et al., 2014; Li et al., 2020b; Lin et al., 2021). Furthermore, the biomaterials utilized *in vivo* ineluctably contact and interact with the red blood cells which play important physiological functions and are also the most abundant blood cells. Herein, the interaction between the red blood cells and CaCO_3_ NRs and the cytotoxicity were used to evaluate the biocompatibility. The results showed that the hemolysis rates of the CaCO_3_ NRs were less than 5% (Figure 4A) within the concentration range from 10 to 200 μg/ml, demonstrating excellent hemocompatibility. In addition, the cytotoxicity of CaCO_3_ NRs was evaluated with the C2C12 cells and L929 cells. The results showed that all the concentrations had cell viability rates of more than 80% (Figure 4B) after coincubating for 24 and 48 h. Therefore, all the results demonstrated that the prepared nanocarriers of the CaCO_3_ NRs had shown no obvious toxicity.

### 3.4 Cellular Internalization Efficiency

**FIGURE 5:**
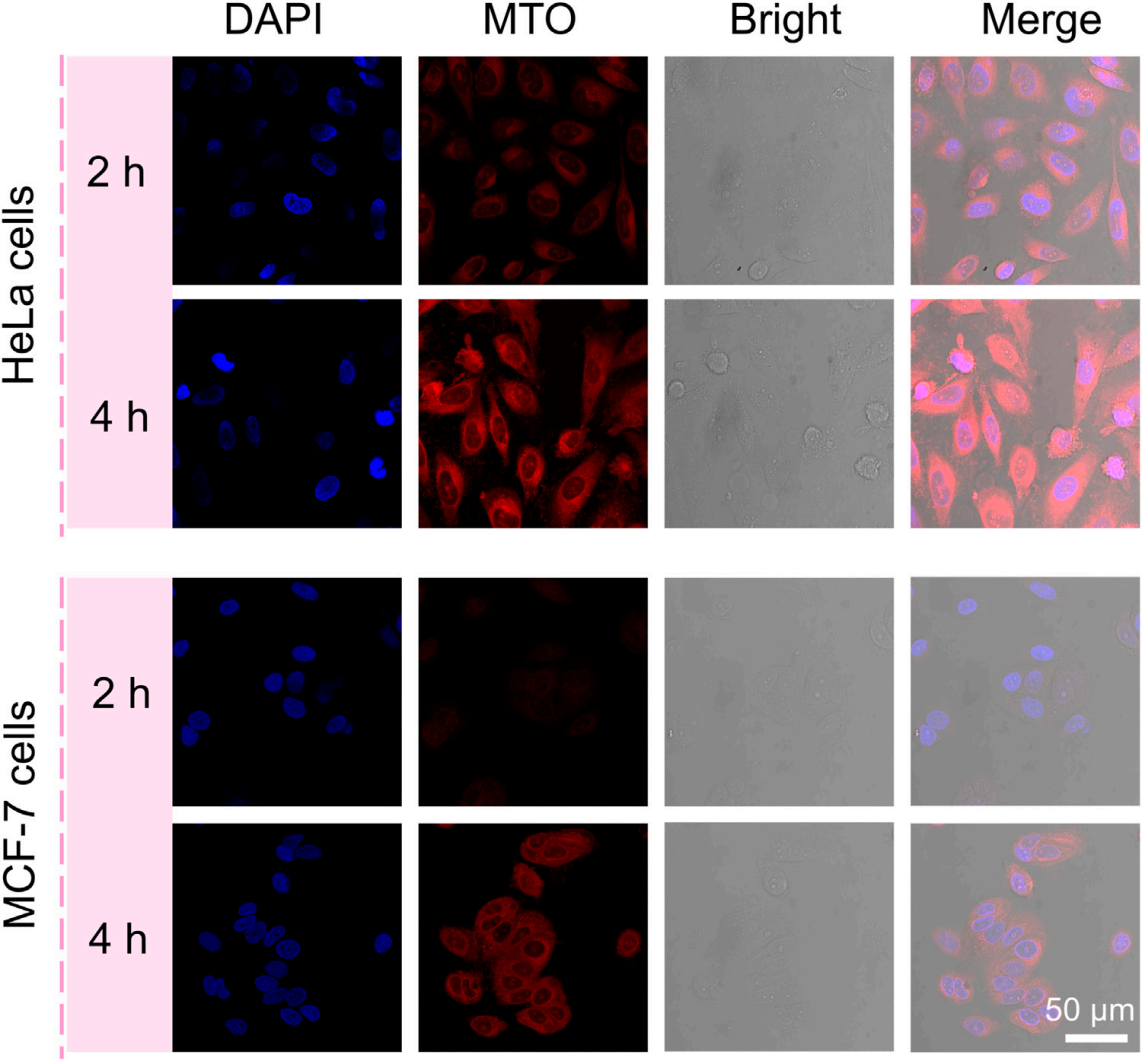
CLSM images of the HeLa cells and MCF-7 cells incubated with MC NRs for 2 or 4 h showing the internalization efficiency of the MC NRs, where the nuclei were counterstained with DAPI.

Based on these rod anisotropic nanoplatforms, next, we studied the interactions of MC nanoparticles *in vitro* with tumor cells. The MCF-7 cells and HeLa cells were incubated with the MC NRs for 2 and 4 h, respectively, at 37°C for observing the cellular uptake ability of our nanoparticles and then imaged by a CLSM (Figure 5). The nucleus of the HeLa cells and MCF-7 cells were stained with DAPI dye with blue fluorescence. Then, red MTO fluorescence emerged inside the HeLa and MCF-7 cells after incubation with the MC NRs, describing the efficient cellular uptake of our nanoparticles. Especially, while MTO fluorescence was observed only in HeLa cell cytoplasm after 2 h, MTO fluorescence appeared in nuclei after 4 h, indicating the dissociation of MC NRs (lysosomes with acid pH). Moreover, the red fluorescence of the MCF-7 cells was weaker than that of the HeLa cells, suggesting that there was a difference in the cellular uptake of the MC NRs, maybe due to the difference in cellular growth. In addition, the design of the nanocarriers requires a good understanding of the mechanisms of cellular uptake which is related to the improvement of therapeutic efficiency (Ding et al., 2018). The cellular uptake of the MC NRs may mainly be mediated through the clathrin-mediated endocytosis pathway according to other reports (Xie et al., 2017; Ding et al., 2018; Yang et al., 2018). We will further research the endocytosis mechanism of the MC NRs in our future study. In brief, these results indicated that the MC nanoparticles were capable of pH-sensitive manner for drug release specifically responding to the acid microenvironment.

### 3.5 *In vitro* Antitumor Performance

**FIGURE 6:**
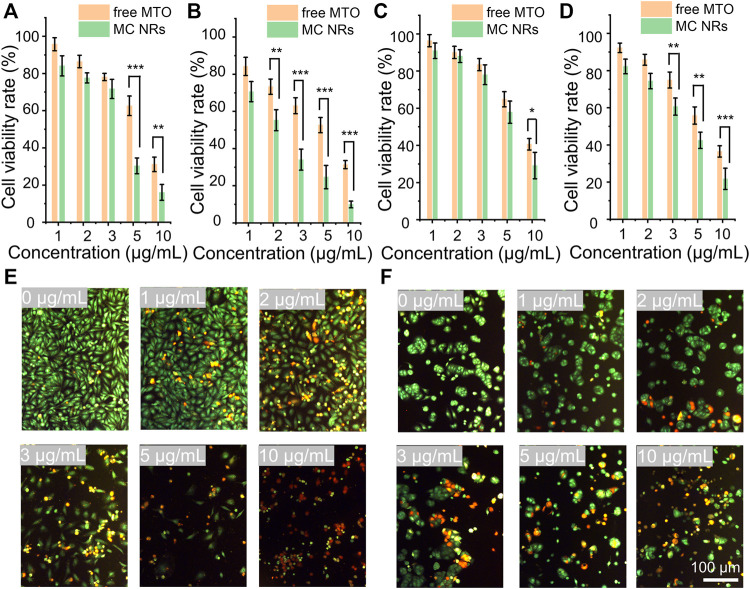
*In vitro* antitumor performance. The cell viability rate of the HeLa cells treated with free MTO and MC NRs for **(A)** 24 h and **(B)** 48 h. The cell viability rate of the MCF-7 cells treated with free MTO and MC NRs for **(C)** 24 h and **(D)** 48 h. Fluorescence images showing **(E)** HeLa cells and **(F)** MCF-7 cells stained with AO (live cells, green fluorescence) and EB (dead cells, red fluorescence) after culturing with different concentration of MTO-CaCO3 NRs (in terms of MTO 0, 1, 2, 3, 5, and 10 μg/ml) for 24 h.

To observe the antitumor performance of the MC NRs *in vitro*, herein, the HeLa human cervical carcinoma cells and MCF-7 human breast cancer cells were set as model cells. Comparing the antitumor effects, the free MTO and MC NRs were treated with the cancer cells in terms of cell viability over 24 and 48 h. First, the HeLa cells and MCF-7 cells were incubated with free MTO or MC NRs at the equivalent MTO concentrations. After coincubating for 24 h or 48 h, the relative cell viabilities were observed by MTT assay (Figures 6A–D). The results show that the MC NRs present a comparable antitumor efficiency to that of free MTO. The cell viability rates of the HeLa cells treated with MC NRs were 30.3 ± 4.2% and 24.7 ± 6.3% with the concentration of 5 μg/ml after 24 and 48 h, comparing 62.6 ± 5.3% and 52.7 ± 4.1% with the free MTO. The cell viability rates of the MCF-7 cells treated with the MC NRs were 29.1 ± 7.1% and 21.8 ± 5.7% with the concentration of 10 μg/ml after 24 and 48 h, comparing 40.6 ± 3.1% and 36.5 ± 3.1% with the free MTO. In addition, the antitumor efficacy of the MC NRs presented a concentration-dependent manner. As expected, the pH sensitivity of the MC NRs after uptake by the tumor cells determines the prominent antitumor effects in the intracellular acid lysosomal environment (Wang et al., 2018b). Then, the abundance of the MTO released from the MC nanoparticles slowly permeates into the nuclei (Figure 5). Meanwhile, the dysfunctional tumor nuclei were induced by the cytotoxicity of the released MTO. With the accumulated cytotoxicity of the MTO, significant tumor cell death was observed at the concentration of 5 and 10 μg/ml for 24 and 48 h.

To further intuitively observe the anti-tumor efficacy of MC NRs, HeLa and MCF-7 cells were stained using the AO/EB kit after treatment with MC NRs for 24 h. Then, the live cells were stained with green fluorescence, early-apoptosis cells were stained with orange fluorescence, and late-apoptosis cells were stained with red fluorescence (Figures 6E,F). Notably, many apoptotic HeLa cells and MCF-7 cells were detected above 3 μg/ml MTO, and the cellular density of both cells significantly decreased with the increase of the concentration of the MC NRs. In addition, the MC NRs exhibit concentration-dependent anticancer activity corresponding to the MTT assay results. Importantly, the MCF-7 cells had fewer late apoptotic cells than HeLa cells, possibly because the MCF-7 cells have a habit of growing into a mass, which is difficult for the MC NRs to penetrate (Hattangadi et al., 2004; Ziegler et al., 2014).

## 4 Conclusion

In summary, the rod anisotropic CaCO_3_ nanoparticles with different aspect ratios were successfully synthesized using fucoidan as a crystal mediator, which adsorbed the calcium ions through sulfate groups. The CaCO_3_ NRs present good biocompatibility, which was suitable for drug delivery. Furthermore, the CaCO_3_ NRs possessed precise pH responsiveness for anticancer-drug release at the tumor microenvironment. In *in vitro* antitumor performance, the MC NRs showed good antitumor effect and cellular uptake for the MCF-7 cells and HeLa cells. Our work provides a new design for forming rod CaCO_3_ nanocarriers. Our findings open the new possibility to prepare calcium carbonate nanoparticles of different shapes with well-tuned structures using other biomacromolecules or polymers.

## Data Availability

The original contributions presented in the study are included in the article/Supplementary Material, further inquiries can be directed to the corresponding authors.
