# Supplementary material for: Fucoidan-Mediated Anisotropic Calcium Carbonate Nanorods of pH-Responsive Drug Release for Antitumor Therapy
Source: Front Bioeng Biotechnol. 2022 Apr 13;10:845821. doi: 10.3389/fbioe.2022.845821 (PMC9043484; doi:10.3389/fbioe.2022.845821)
Supplement: Supplementary file 1 [file DataSheet1.DOCX]

*Supporting information*

# Fucoidan-mediated anisotropic calcium carbonate nanorods of pH-responsive drug release for antitumor therapy


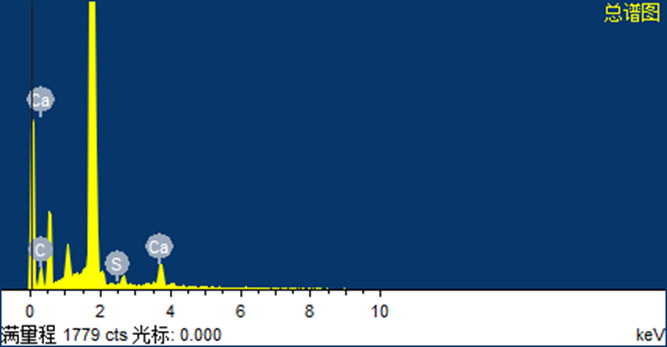


**Figure S1.** Energy dispersive spectrometer (EDS) of CaCO_3_ NRs.


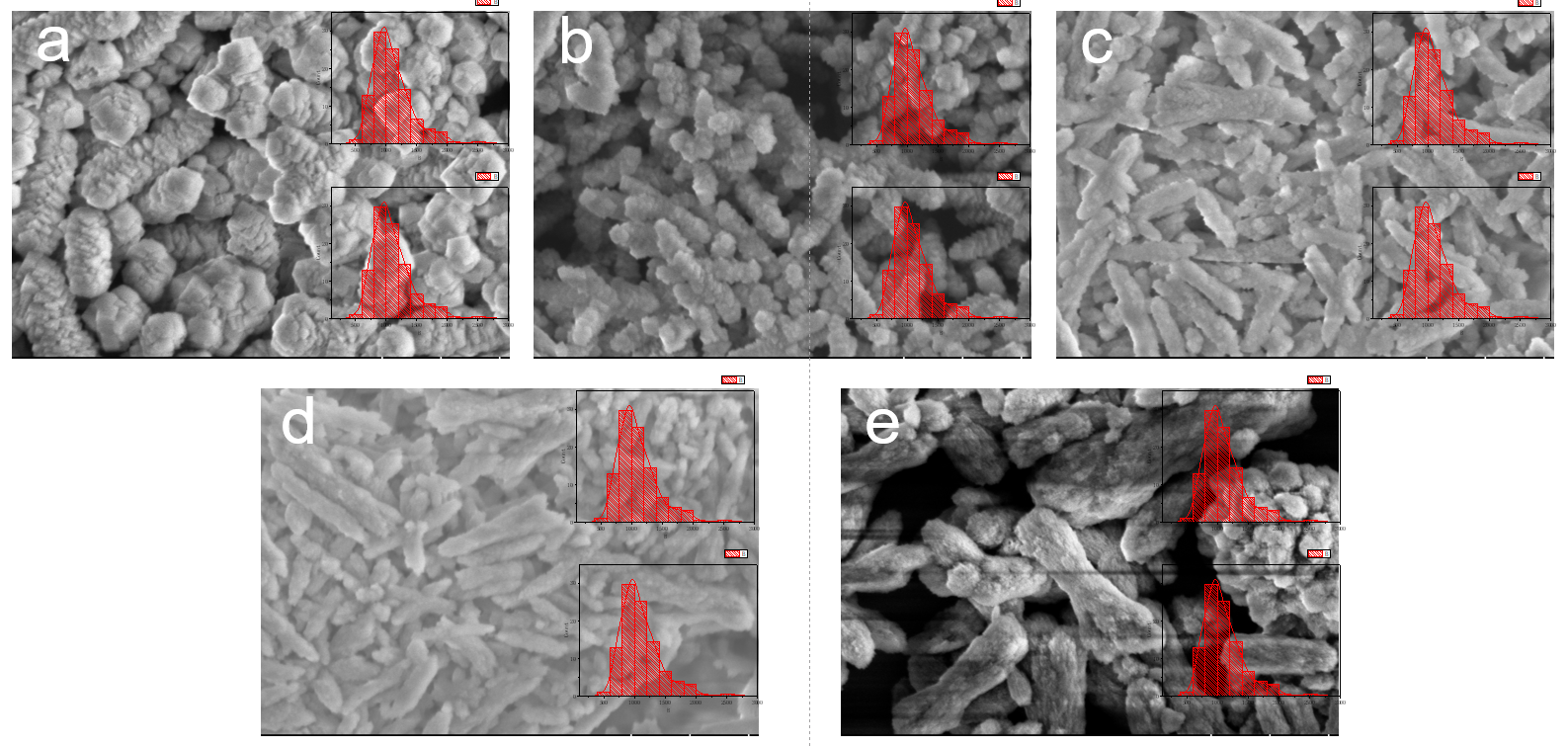


**Figure S2.** SEM images of CaCO_3_ NRs with various concentration of fucoidan (a) 5, (b) 10, (c) 20, (d) 40, (d) 50 µg/mL





**Figure S3.** Calibration curve of MTO.
